# The impact of an educational intervention on neonatal intensive care unit nurses’ knowledge, attitudes, and perceptions of parental participation in kangaroo mother care

**DOI:** 10.1371/journal.pone.0306888

**Published:** 2024-08-01

**Authors:** Sawsan Abuhammad, Roaa Karimeh, Alia Mahadeen

**Affiliations:** 1 Maternal and Child Health Nursing Department, School of Nursing, Jordan University of Science and Technology, Irbid, Jordan; 2 Department of Maternal Child Health Nursing, School of Nursing, The University of Jordan, Amman, Jordan; Bingol University: Bingol Universitesi, TURKEY

## Abstract

**Aim:**

The aim of this study is to evaluate the effectiveness of the education interventions on NICU nurses’ knowledge, attitudes, and perceptions of parental participation in kangaroo mother care (KMC) in NICU.

**Method:**

A quasi-experimental pretest/posttest nonequivalent group design was used to evaluate the effectiveness of the education interventions on NICU nurses’ knowledge, attitudes and perceptions of parental participation in KMC delivered in the neonatal intensive care units at three hospitals. 160 nurses participated in the study, with a division of 80 in the educational group, and 80 in the non-educational group.

**Results:**

The results showed that the educational group was statistically different in knowledge post-intervention (t = -2.819, df = 79, p≤0.001). The pre-intervention mean attitude for the educational group was 19.81 (SD = 4.3). The findings expressed that the educational group was statistically different in attitude in the post-intervention phase (t = -3.66, df = 79, p≤0.001). The results reflect a significant effect in the educational group regarding nurses’ perspectives of parental participation post-intervention (t = 5.496, df = 79, p≤0.001).

**Conclusion:**

Our findings showed that providing nurses with additional education increased their knowledge, improved their attitudes, and enhanced their perceptions of parental support in NICU. Providing staff with an educational intervention about KMC in NICU can enhance nursing knowledge, attitudes, and perceptions of parental participation in neonate care in the NICU.

## Introduction

Low birth weight neonates represent a major critical problem for the neonate population in both developed and developing countries [1]. Low birth weight poses significant health risks to neonates and negatively affects their quality of life during infancy [2]. Nonetheless, recently updated literature had proven that kangaroo mother care (KMC) is an effective alternative procedure that may be helpful in the improvement of physiologic changes alongside decreasing the negative outcomes in NICU neonates [3, 4].

KMC is reported in literature as an essential strategy in the care of premature neonates. Its use has been highly recommended thus far in resource-limited countries and settings. Its implementation and the resulting high level of outcomes may result in decreasing the mortality rates by around 40% [5]. Neonatal health problems associated with low-birth weight neonates, such as sepsis, can be prevented by KMC and breastfeeding, as the literature confirms [6, 7]. Moreover, in the previously mentioned locations, such as South Asia, KMC has been widely studied and confirmed to bring about huge health developments. It is found to reduce morbidity rates by being effective in fighting infections and nosocomial infections, decreasing lower respiratory tract infections, and decreasing the length of hospital stays [8, 9].

The KMC has been shown to decrease neonatal mortality. Also, KMC is a simple and low-cost intervention that can have a significant impact on reducing neonatal mortality, especially in resource-limited settings [10, 11]. However, successful implementation of KMC requires a supportive healthcare environment and adequate training and resources for healthcare providers, nurses, and parents [12, 13].

Neonatal nurses play a huge role in practicing KMC in NICUs, and they are responsible for easing and facilitating the mothers’ and families’ awareness [14–18], as well as creating positive perceptions of KMC. It should be considered that increasing nursing knowledge may result in even more positive outcomes of KMC practice, as widely reported in the literature. Additionally, increasing nurses’ knowledge can have a significant positive effect on their attitudes toward KMC [19].

While KMC reflects prominent benefits, several research studies point out some barriers associated with the practice [13]. Generally, health issues related to health systems, inapplicable modalities, and lack of health-care knowledge are reported. In addition, lack of time and space, limited staff contact with families, inadequate knowledge of staff in family motivation, and social cultural norms remain major obstacles to the best KMC practice [20]. A targeted education program should enhance knowledge and positive attitudes, ensure safe and effective implementation, facilitate collaboration and teamwork, and promote advocacy for KMC [21].

Policy makers need to focus their attention on creating comprehensive education interventions in the area of research and implementing it in the relevant settings [22, 23]. Having trained nurses present the education interventions to mothers during pregnancy may result in more effective practice due to improved attitudes and perceptions of KMC [24, 25]. The literature is full of alternative solutions that are presented as possible interventions to be implemented in various sites and settings. The aim of this study is to determine the impact of the education interventions on NICU nurses’ knowledge, attitudes, and perceptions of parental participation in KMC in NICU.

## Method

### Design

A quasi-experimental pretest/posttest nonequivalent groups design was used to evaluate the effectiveness of educational interventions on nurses in Jordan that include knowledge, attitudes, and perceptions toward parental participation in KMC. This design is considered feasible in relation to the possibility of real experimentation in a neutral environment. This design may motivate the researchers to use it such as: (1) low in the cost which require fewer courses and take less time, (2) it can be more generalization because it can conduct in the real world(3) and the researchers can view the data retrospectively and prospectively.

### Sample and sampling

The nurses who worked at King Abdullah University Hospital were placed in the interventional group. All NICU nurses in Princess Raya Hospital were put into the non-educational group. The nurses from Al Bashir Hospital were divided between the two groups. Based on G power, an adequate sample size for two nonequivalent group pre-post designs with a power level 0.9, level of 0.05, and effect size of 0.3 was considered to be 75 participants for each group. To recruit the participants, a convenience sampling technique was used. To fit the eligibility criteria, each candidate had to be a registered nurse who directly cares for neonates, able to communicate in both Arabic and English, willing to participate in the study, and hold a bachelor’s degree or higher educational level. Candidates were also required to have no previous experience in KMC or any related course.

### Setting

The settings are northern and central regions of Jordan and focused on three hospitals: Princess Raya Hospital (governmental hospital), Al-Bashir Hospital (governmental hospitals), and King Abdullah University Hospital (a teaching hospital). These settings were chosen because the nurses from the three hospitals are well educated with bachelor’s or postgraduate degrees. In addition, these hospitals offer continuous education courses that improve the knowledge, attitudes, and perceptions of staff, and more specifically in the NICU. Moreover, educational courses for parenting support are still uncommon courses in the hospitals. The instrument used in the study is composed of four parts: demographic information, nurses’ level of knowledge of KMC, nurses’ attitudes regarding KMC, and nurses’ perception of parental participation in KMC. The following paragraphs describe each part separately, involving entire questions:

### Demographics

This part includes demographic information related to sample characteristics that were previously involved in the literature.

### Nurses’ knowledge of KMC

This instrument was used to assess the nurse’s KMC in the NICU, considering their entire experience in nursing as well as their experience in the neonatal care units. The instrument was translated from the English version of KMC [26]. The instrument consists of 17 items about knowledge of benefits (7 items), situation (6 items), and appropriate use of KMC (4 items). Nine items were reverse scored (items 2, 4, 5, 8, 11, 12, 15, and 17). The chosen 17 questions were assumed to represent reported literature information about KMC to achieve validation points of the knowledge set. In fact, the literature information was the principal component for this part of the questionnaire addressing nurse and parent-related considerations. Indeed, items were represented to achieve specific details of how and when KMC practice could be performed. The questions were designed to be easily comprehended by the nurses, even if they are not highly educated. All items required a response of (Correct = 1, or Incorrect = 0). Cronbach’s alpha is 0.80. The reliability reported by Engler et al. [26] was 0.84.

### Nurses’ attitudes toward KMC

This part was used to assess the nurses’ attitudes toward KMC using the Pauline Chia scale for attitude [27]. The statements of this instrument were formulated to cover participants’ attitudes toward KMC alongside the benefits desired from this kind of care in the neonatal settings. The scale consists of 14 items that use the 5-point Likert scale, 5 items describing benefits, 3 items for current practice, and 6 items about the role of the nurse in KMC. A total highest score of 70, and lowest score 14. Cronbach’s alpha values range from 0.86 to 0.92.

### Nurses’ perceptions of parental participation in KMC

This tool was used to measure nurses’ perceptions of parental participation in KMC. This instrument consists of five items that focus on the importance of parents participating in KMC and the nurse’s role in providing information about KMC. The items are phrased as follows: 1. Nurses should encourage parents to come to the NICU; 2. KMC is something I discuss with parents that they can do for their newborn; 3. I introduce KMC to mothers on the first day in the NICU; 4. KMC is not harmful; 5. Nurses should be supportive in the provision of KMC. All responses were either (Yes, No). The highest score is 5 and the lowest score is zero. The Cronbach’s alpha of the instrument is .82.

### The intervention

The aim of this program was to educate NICU nurses for increased knowledge, and improved attitudes and perceptions of parental participation in KMC. The program gives some information to help nurses understand the process of implementing KMC and to get the process moving. Nurses are urged to motivate parents to engage in KMC, but the program does not attempt to provide answers or guidelines for everything that should be done. The target users are mainly staff of healthcare facilities, although we also included a short section for officials in health departments or ministries. This program includes two main parts, as described in the next section.

First part includes information for parents/mothers: including definition of KMC, benefits of KMC, presence of advantages and disadvantages, skin-to-skin contact, equipment needed in KMC, time duration of KMC, frequency of KMC, allowance of breastfeeding, allowance of clothes cleaning, the typical neonate hygiene, and typical maternal hygiene. In addition, this part includes components of KMC (position of KMC, nutrition, discharge teaching, and support needed in mothers’ encouragement.

Second part include implementation of position of KMC and Science of implementation of the kangaroo position: these parts discuss and demonstrate the ideal contact/position of KMC, which has several choices; to place the neonate between mother’s breasts, or chest to chest in a reclined or semi-recumbent position. Mother also taught how she secures her neonate by blinder, with turning head to one side in slightly extended position and placing the tip of blinder at just below ears. Mothers learn to avoid head forward flexion and hyperextension, which protects airway patency and allows eye-to-eye contact with her neonate.

### Data collection procedure

Prior to commencing data collection, approval from IRB was done. Additionally, permission was sought from the respective hospital managers. The principal investigator conducted meetings with the heads of departments in selected Neonatal Intensive Care Units (NICUs), elucidating the study’s objectives and data collection procedures. Collaborating with department heads, suitable times and locations were determined to meet with nurses, who were subsequently invited to participate in the study. Lists containing the names of nurses and their respective shift schedules were acquired from each department. Nurses on three different shifts were approached during their working hours, provided with an explanation of the study’s purpose and data-collection procedures, and requested to sign an informed consent. Nurses were assured that their responses would be treated confidentially, aggregated in group reports, and that no personally identifying information would be disclosed. Those who agreed to participate were given the opportunity to complete a self-reported questionnaire at their convenience, returning it in a sealed envelope to the head of the department’s office. The researcher collected the completed questionnaires, and participants were informed that they could contact the authors for any further clarification or questions. The questionnaire typically took 10–15 minutes to complete. Data collection occurred between March 1, 2023, and May 5, 2023.

### Interventional group

Some KAUH NICU nurses (N = 20) and some Al-Bashir NICU nurses (N = 60) were placed in the interventional group. After informed consent was obtained from the participants, they were requested to fill out the pre-test questionnaire to assess baseline level which was described in the instrument section previously. The time and place set for the educational sessions were determined according to the nurses’ preference. Subsequently, the educational sessions were presented to participants via laptop PowerPoint presentation, in addition to via the WhatsApp application. The participants filled out the same questionnaire for post-test after receiving the educational sessions.

### Non-educational group

All NICU nurses who worked in Raya Hospital (N = 11) and some NICU nurses from Al-Bashir Hospital (N = 46) were placed in the non-educational group. After getting the informed consent from the participants, they were requested to fill out the questionnaire for the first time to take baseline level. They filled out the same questionnaire for a second time three days later after the nurses had the opportunity to search about KMC in books or online sources at the same time as the interventional group. Also, the educational sessions used in the educational group were sent to the non-educational group after completion of the data collection from them.

### Ethical consideration

Approval was obtained from responsible IRBs. The study was conducted with complete awareness of the preservation of human rights, due to the fact that the study shared private information about the participants. The participating nurses received full information about the study, and they signed the informed consent form. Also, to ensure the confidentiality and anonymity of participants, they were identified by a number rather than their names. Participants were informed that there was no direct benefit to them in the form of compensation for their involvement in the study.

### Data analysis

The data entered to SPSS was checked for accuracy and then the statistical analysis was done. The continuously measured variables were described by using the means, the median, the standard deviation and the minimum and maximum, and the frequencies and percentage were used to describe the categorical factor. A Pearson chi-squared test was used to evaluate the categorical variables association and the bivariate correlation between metric continuous variables, and the independent variables were analyzed by the Pearson’s correlation test. In addition, the bivariate chi-squared test was used to compare the baseline between the nurses in the interventional groups and non-educational group. The alpha significance level was considered at 0.050. The mixed linear models’ analysis of repeated measures was performed to assess the statistical significance of the effect of the intervention (the educational program) on nurses’ knowledge, attitudes, and perceptions of parents’ participation in KMC between the pre- and post- times as a repeated measure vector. All statistical tests were two-tailed.

## Results

### Participants characteristics

The chi-square test was performed to compare the ratio of the categorical variables for the demographic data between the two groups (control and educational groups), the finding indicated that the nurses in the interventional group did not differ significantly in Workplace (p>0.05) of those in the non-educational group. For more details about demographic characteristics and nursing study groups in Table 1.

**Table 1:** Comparison of the nurses’ socio-demographic and professional characteristics between the two groups of nurses. N = 160.

		Non-educational group	Interventional group	*χ*^2^/df	p-value
**Age**	22–30	28(17.5)	33(20.625)	0.9775	0.376
	31–40	48(30)	40(25)		
	41–50	4(2.5)	7(4.375)		
**marital status**	married	52(32.5)	46(28.75)	1.74	0.156
	single	21(13.125)	26(16.25)		
	widow	3(13.125)	0		
	divorced	4(2.5)	8(5)		
**Do you have children?**	Yes	50(31.25)	47(29.375)	0.236	0.627
	No	30(18.75)	33(20.625)		
**Workplace**	King Abdullah University Hospital	23(14.375)	20(12.5)	6.525	**≤0.0011**
	Princess Raya Hospital	11(6.875)	0(0.0)		
	Al-Bashir Hospital	46(28.75)	60(37.5)		
**Years of Experience**	<5	14(8.75)	20(12.5)	0.67	0.51
	5–10	45(28.125)	41(25.625)		
	11–20	21(13.125)	19(11.875)		
**Number of years of experience in pediatric departments**	<5	14(8.75)	20(12.5)	0.931	0.628
	5–10	45(28.125)	41(25.625)		
	11–20	21(13.125)	19(11.875)		
**Educational level**	Bachelor’s	66(41.25)	71(44.375)	1.26	0.26
	Master’s	14(8.75)	9(5.625)		
**Received an introductory workshop on KMC before**	Yes	0(0.0)	1(0.625)	1	0.316
	No	80(50)	79(49.375)		
**Worked in a department providing KMC**	No	160(100)	160(100)	-	-

### The differences in the interventional group between pre-test in the post test score of knowledge of KMC

The findings of the t-test expressed that the educational group was statistically different in knowledge (t = -2.819, df = 79, p≤0.001). The chi-square test was conducted to compare the knowledge in the two groups. Nurses in both groups have been asked about 17 items that assessed parental participation. The result indicated that the nurses in the two groups had differences significantly in their response **except** item (5,8,10,11,15,16,17) in detail, See Table 2.

**Table 2:** Comparison of nurses’ attitudes toward parental participation in the post-pre intervention time.

		Pre-intervention, n = 80	Post- intervention, n = 80	*χ*^2^/df = 1	P-value
1	Babies appear to be contented in KMC.			10.09	≤0.001
	Yes, n (%)	64(80)	77(96.3)		
	No, n (%)	16(20)	3(3.8)		
2	Babies on oxygen therapy experience a decrease in oxygen saturation.			15.63	≤0.001
	Yes, n (%)	32(40)	10(12.5)		
	No, n (%)	48(60)	70(87.5)		
3	Babies on phototherapy can participate in KMC.			39.83	≤0.001
	Yes, n (%)	46(57.5)	79(98.8)		
	No, n (%)	34(42.5)	1(1.3)		
4	Babies on vasopressors should NOT engage in KMC.			32.31	≤0.001
	Yes, n (%)	36(45)	70(87.5)		
	No, n (%)	44(55)	10(12.5)		
5	Babies typically experience more bradycardic episodes during KMC.			1.34	**0.25**
	Yes, n (%)	32(40)	25(31.3)		
	No, n (%)	48(60)	55(68.8)		
6	Babies with peripheral IVs can participate in KMC.			14.68	≤0.001
	Yes, n (%)	60(75)	77(96.3)		
	No, n (%)	20(25)	3(3.8)		
7	KMC has been shown to improve breathing patterns in preterm neonates by reducing apnea.			9.04	≤0.001
	Yes, n (%)	62(77.5)	78(97.5)		
	No, n (%)	18(22.5)	2(2.6)		
8	KMC is contraindicated in neonates less than 28 weeks gestation.			≤0.001	**1.00**
	Yes, n (%)	58(72.5)	58(72.5)		
	No, n (%)	22(27.5)	22(27.5)		
9	KMC is contraindicated in neonates weighing less than 1000 grams.			44.01	≤0.001
	Yes, n (%)	52(65)	11(13.8)		
	No, n (%)	28(35)	69(86.3)		
10	KMC is now considered safe as an alternative approach to care for medically stable, continuing care preterm neonates.			0.25	**0.62**
	Yes, n (%)	70(87.5)	72(90)		
	No, n (%)	10(12.5)	8(10)		
11	Most neonates experience a decrease in temperature during KMC.			0.10	**0.75**
	Yes, n (%)	36(45)	38(47.5)		
	No, n (%)	44(55)	42(52.5)		
12	Published reports of clinical observations indicate that the rate of accidental intubating is higher with KMC than with traditional methods of holding.			13.09	≤0.001
	Yes, n (%)	40(50)	62(77.5)		
	No, n (%)	40(50)	18(22.5)		
13	Research has indicated that neonates who receive KMC increase their mother’s milk supply.			8.01	0.01
	Yes, n (%)	61(76.3)	74(92.5)		
	No, n (%)	19(23.8)	6(7.5)		
14	Research indicates that KMC promotes quiet sleep.			3.84	0.05
	Yes, n (%)	63(78.8)	72(90)		
	No, n (%)	17(21.3)	8(10)		
15	Research shows that neonates with arterial lines should NOT engage in KMC.			0.90	**0.34**
	Yes, n (%)	45(56.3)	39(48.8)		
	No, n (%)	35(43.8)	41(51.2)		
16	The most physiologically stressful part of KMC for the neonate is the transfer to the parent’s chest.			1.41	**0.24**
	Yes, n (%)	51(63.7)	58(72.5)		
	No, n (%)	29(36.3)	22(27.5)		
17	There is an increased risk of infection in the neonate with KMC.			0.48	**0.49**
	Yes, n (%)	54(67.5)	58(72.5)		
	No, n (%)	26(32.5)	22(27.5)		

### The differences in the interventional group between pre-test in the post test score of attitudes of KMC

The findings of the paired t-test expressed that the educational group was statistically different in attitude the second time (t = -3.66, df = 79, p≤0.001). The Chi-square was conducted to compare the attitude of the same group before and after attending educational program. The result indicated that the attitude of nurses showed significant differences in their item of instrument **except** item 10. See Table 3.

**Table 3:** Comparison of nurses’ attitudes toward parental participation in the post-pre intervention time.

		Pre-intervention, n = 80	Post- intervention, n = 80	*χ*^2^/df = 4	P-value
1	Kangaroo care should not be practiced with an intubated infant.			11.02	≤0.001
	Strongly disagree, n (%)	24(30)	3(3.8)		
	Disagree, n (%)	10(12.5)	6(7.5)		
	Neutral, n (%)	16(20)	2(2.5)		
	agree, n (%)	19(23.8)	38(47.5)		
	Strongly agree, n (%)	11(13.8)	31(38.8)		
2	Kangaroo care should only be practiced for infants weighing 1000 gm or more.			9.75	≤0.001
	Strongly disagree, n (%)	15(18.8)	4(5)		
	Disagree, n (%)	9(11.3)	1(1.3)		
	Neutral, n (%)	20(25)	3(3.8)		
	agree, n (%)	12(15)	34(42.5)		
	Strongly agree, n (%)	24(30)	38(47.5)		
3	Kangaroo care should begin within a few hours of birth.			8.91	≤0.001
	Strongly disagree, n (%)	17(21.3)	44(55)		
	Disagree, n (%)	34(42.5)	33(41.3)		
	Neutral, n (%)	22(27.5)	2(2.5)		
	agree, n (%)	4(5)	1(1.3)		
	Strongly agree, n (%)	3(3.8)			
4	All parents should be encouraged to practice KMC.			3.79	≤0.001
	Strongly disagree, n (%)	32(40)	54(67.5)		
	Disagree, n (%)	37(46.3)	24(30)		
	Neutral, n (%)	8(10)	1(1.3)		
	agree, n (%)	2(2.5)	1(1.3)		
	Strongly agree, n (%)	1(1.3)	0		
5	All parents should be given relevant information on KMC.			2.85	0.04
	Strongly disagree, n (%)	48(60)	62(77.5)		
	Disagree, n (%)	24(30)	17(21.3)		
	Neutral, n (%)	6(7.5)	1(1.3)		
	agree, n (%)	2(2.5)	0		
	Strongly agree, n (%)	(0)	0		
6	Nurses should remain with parents for participation and assistance during Kangaroo care.			7.14	≤0.001
	Strongly disagree, n (%)	32(40)	47(58.8)		
	Disagree, n (%)	19(23.8)	24(30)		
	Neutral, n (%)	5(6.3)	9(11.3)		
	agree, n (%)	22(27.5)	0		
	Strongly agree, n (%)	2(2.5)	0		
7	Nurses should facilitate KMC when the NICU is quiet.			6.89	≤0.001
	Strongly disagree, n (%)	33(41.3)	51(63.7)		
	Disagree, n (%)	19(23.8)	24(30)		
	Neutral, n (%)	26(32.5)	5(6.3)		
	agree, n (%)	2(2.5)	0		
	Strongly agree, n (%)	0	0		
8	Facilitating KMC is professionally satisfying.			7.49	≤0.001
	Strongly disagree, n (%)	31(38.8)	50(62.5)		
	Disagree, n (%)	31(38.8)	30(37.5)		
	Neutral, n (%)	14(17.5)	0		
	agree, n (%)	4(5)	0		
	Strongly agree, n (%)	0	0		
9	Facilitating KMC is an added burden to NICU nurses.			2.44	0.05
	Strongly disagree, n (%)	15(18.8)	20(25)		
	Disagree, n (%)	34(42.5)	32(40)		
	Neutral, n (%)	13(16.3)	22(27.5)		
	agree, n (%)	8(10)	4(5)		
	Strongly agree, n (%)	10(12.5)	2(2.5)		
10	Kangaroo care promotes bonding.			1.54	**0.21**
	Strongly disagree, n (%)	51(63.7)	61(76.3)		
	Disagree, n (%)	25(31.3)	17(21.3)		
	Neutral, n (%)	4(5)	2(2.5)		
	agree, n (%)	0	0		
	Strongly agree, n (%)	0	0		
11	Kangaroo care has a positive effect on physical wellbeing of infant.			9.92	≤0.001
	Strongly disagree, n (%)	46(57.5)	69(86.3)		
	Disagree, n (%)	23(28.7)	11(13.8)		
	Neutral, n (%)	11(13.8)	0		
	agree, n (%)	0	0		
	Strongly agree, n (%)	0	0		
12	Kangaroo care enhances the parents’ confidence.			6.68	≤0.001
	Strongly disagree, n (%)	43(53.8)	63(78.8)		
	Disagree, n (%)	32(40)	17(21.3)		
	Neutral, n (%)	5(6.3)	0		
	agree, n (%)	0	0		
	Strongly agree, n (%)	0	0		
13	Kangaroo care results in more effective breastfeeding.			4.03	≤0.001
	Strongly disagree, n (%)	47(58.8)	66(82.5)		
	Disagree, n (%)	19(23.8)	13(16.3)		
	Neutral, n (%)	4(5)	1(1.3)		
	agree, n (%)	8(10)	0		
	Strongly agree, n (%)	2(2.5)	0		
14	Potential benefits of KMC have been overstated.			10.27	≤0.001
	Strongly disagree, n (%)	41(51.2)	72(90)		
	Disagree, n (%)	27(33.8)	8(10)		
	Neutral, n (%)	11(13.8)	0		
	agree, n (%)	0	0		
	Strongly agree, n (%)	1(1.3)	0		

### The differences in the interventional group between pre-test in the post test score of nurses perception of parent participation

The findings of paired t-tests expressed that the educational group was statistically different in perception and second time. The results reflect a significant effect in the educational group of nursing perspective (t = 5.496, df = 79, p≤0.001). The chi-square test was conducted to compare the nurses’ perception in the two groups. Nurses in both groups have been asked about 17 items that assessed parental participation. The result indicated that the nurses in the two groups had differences significantly in their responses. See Table 4.

**Table 4:** Comparison of nurses perception questions toward parental participation in the post intervention time.

		Pre-intervention, n = 80	Post- intervention, n = 80	*χ*^2^/df = 1	P-value
1	I should encourage parents to come to the NICU.			8.012	≤0.0015
	Yes, n (%)	61(76.3)	74(92.5)		
	No, n (%)	19(23.8)	6(7.5)		
2	KMC is something I discuss with parents that they can do for their neonate.			17.936	≤0.001
	Yes, n (%)	49(61.3)	72(90)		
	No, n (%)	31(38.8)	8(10)		
3	I introduce KMC to mothers on the first day in the NICU.			9.477	≤0.0012
	Yes, n (%)	51(63.7)	68(85)		
	No, n (%)	29(36.3)	12(15)		
4	KMC is not harmful.			11.841	≤0.0011
	Yes, n (%)	40(50)	61(76.3)		
	No, n (%)	40(50)	19(23.8)		
5	Nurses should be participation in provision of KMC.			8.041	≤0.0015
	Yes, n (%)	58(72.5)	72(90)		
	No, n (%)	22(27.5)	8(10)		

### Correlation between attitude, knowledge, and nurses perception toward parents participation in KMC in non-educational group

The yielded analysis findings of Pearson correlation suggested that the variables are correlated significantly between pretest knowledge and attitude of KMC (r^2^ = 0.205, p<0.050). Also, these variables significantly decreased after intervention (r = .22, p<0.050). See Table 5.

**Table 5:** Bivariate Pearson’s correlations between attitude, knowledge, and nurses perception toward parents participation in KMC in non-educational group.

		attitude (pre)	attitude (post)	knowledge (pre)	knowledge (post)	nursing vision (pre)	nursing vision (post)
attitude (pre)	Pearson Correlation	1					
	Sig. (2-tailed)						
attitude (post)	Pearson Correlation	0.037	1				
	Sig. (2-tailed)	0.743					
knowledge (pre)	Pearson Correlation	0.138	-0.039	1			
	Sig. (2-tailed)	0.221	0.729				
knowledge (post)	Pearson Correlation	-0.065	0.220	0.191	1		
	Sig. (2-tailed)	0.567	0.050	0.090			
nursing Perception (pre)	Pearson Correlation	-0.025	**.358** ^**^	-≤0.0017	0.038	1	
	Sig. (2-tailed)	0.825	≤0.0011	0.949	0.736		
nursing Perception (post)	Pearson Correlation	**.285** ^*^	0.040	0.188	0.037	0.014	1
	Sig. (2-tailed)	0.010	0.726	0.095	0.742	0.902	

### Correlation between attitude, knowledge, and nurses perception toward parents participation in KMC in educational group

The yielded analysis findings suggested the variables KMC are correlated significantly in pretest (r^2^ = 0.308, p<0.050). Also, these variables are correlated significantly posttest (r^2^ = 0.22, p<0.050). See Table 6.

**Table 6:** Bivariate Pearson’s correlations between attitude, knowledge, and nurses perception toward parents participation in KMC in educational group.

		attitude (pre)	attitude (post)	knowledge (pre)	knowledge (post)	nursing vision (pre)	nursing vision (post)
attitude (pre)	Pearson Correlation	1					
	Sig. (2-tailed)						
attitude (post)	Pearson Correlation	0.053	1				
	Sig. (2-tailed)	0.639					
knowledge (pre)	Pearson Correlation	**.299** ^**^	0.129	1			
	Sig. (2-tailed)	≤0.0017	0.255				
knowledge (post)	Pearson Correlation	-0.106	-0.190	**-.301** ^**^	1		
	Sig. (2-tailed)	0.348	0.091	≤0.0017			
nursing Perception (pre)	Pearson Correlation	**.357** ^**^	0.120	**.550** ^**^	-0.141	1	
	Sig. (2-tailed)	≤0.0011	0.290	≤0.0010	0.211		
nursing Perception (post)	Pearson Correlation	0.073	-0.109	0.142	0.051	0.045	1
	Sig. (2-tailed)	0.519	0.335	0.208	0.651	0.695	

### Predictors attitude toward parents participation in in KMC

The interaction effect of (time × intervention) found that nurses in the educational group had a significantly higher mean perception towards parental participation at post educational group perception score in comparison to the nurses who had not received the educational session (β = 2.81, p ≤0.001). The finding showed that the nurses in the educational group had a significantly higher mean perception towards parental participation in general (both, pre and post attitude combined) compared to those nurses in the non-educational group (β = 2.8125, p ≤0.001). However, the change in nurse’s attitude was statistically significant in the nurses who had received the intervention, their pre-test (post, as well as overall perception) exceeded that for those nurses who did not receive the educational session which suggests a significant positive effect of the educational session on the attitude of nurses in general. See Table 7.

**Table 7:** Multivariate mixed effects generalized repeated measures analysis explaining the effect of the educational intervention program on the attitude toward parental participation. N = 320 records.

Parameter	Estimate	Std. Error	df	T	Sig.	95% Confidence Interval	
						Lower Bound	Upper Bound
Intercept	59.075	0.71	316	82.695	**0**	57.67	60.48
[Group = intervention]	-4.53	1.01	316	-4.491	**0**	-6.53	-2.55
[Group = control reference comparison]	0	0	.	.	.	.	.
[time1 = post-test	-3.3	1.01	316	-3.266	**≤0.0011**	-5.29	-1.31
[time2 = pre- test]	0	0	.	.	.	.	.
[Group = intervention] * [time1 = post-test]	2.8125	1.42	316	1.969	**0.05**	≤0.001	5.62
[Group = intervention] * [time2 = pre-test]	0	0	.	.	.	.	.
[Group = control] * [time1 = post-test]	0	0	.	.	.	.	.
[Group = control] * [time2 = pre-test]	0	0	.	.	.	.	.

### Predictors knowledge toward parents participation in in KMC

The interaction effect of (time × intervention) found that nurses in the educational group had a significantly higher mean perception towards parental participation at post educational group perception score in compared to the nurses who had not received the educational session (β = -1, p ≤0.001). The finding showed that the nurses in the educational group had a significantly higher mean perception towards parental participation in general (both, pre and post attitude combined) compared to those nurses in the non-educational group (β = 1.62, p ≤0.001). However, the change in nurse’s knowledge was statistically significant in the nurses who had received the intervention, their pre-test (post, as well as overall perception) exceeded that for those nurses who did not receive the educational session which suggest a significant positive effect of the educational session on the knowledge nurses in general. See Table 8.

**Table 8:** Multivariate mixed effects generalized repeated measures analysis explaining the effect of the educational intervention program on the knowledge toward parental participation. N = 320 records.

parameter	Estimate	Std. Error	df	t	Sig.	95% Confidence Interval	
						Lower Bound	Upper Bound
Intercept	22.13	.272866	316	81.129	.000	21.600	22.67
[Group = intervention]	1.62	.385	316.000	4.211	.000	.865	2.38
[Group = control reference comparison]	0^b^	0	.	.	.	.	.
[time1 = post-test	1.025	.385	316	2.656	.008	.2657	1.78
[time2 = pre- test]	0^b^	0	.	.	.	.	.
[Group = intervention] * [time1 = post-test]	-1.0	.545	316	-1.832	.068	-2.073	.073
[Group = intervention] * [time2 = pre-test]	0^b^	0	.	.	.	.	.
[Group = control] * [time1 = post-test]	0^b^	0	.	.	.	.	.
[Group = control] * [time2 = pre-test]	0^b^	0	.	.	.	.	.

### Predictors nurses perception toward parents participation in in KMC

The mixed linear model was appropriate for the nurses’ nurses Perception scores and was generally statistically significant. The interaction effect of (time × intervention) found that nurses in the educational group had a significantly higher mean perception towards parental participation at post educational group perception score in compared to the nurses who had not received the educational session (β = -0.912, p ≤0.001). The finding showed that the nurses in the educational group had a significantly higher mean perception towards parental participation in general (both, pre and post attitude combined) compared to those nurses in the non-educational group (β = .675, p ≤0.001). However, the change in nurse’s nurses Perception was statistically significant in the nurses who had received the intervention, their pre-test (post, as well as overall perception) exceeded that for those nurses who did not receive the educational session which suggest a significant positive effect of the educational session on the nurses Perception nurses in general. See Table 9.

**Table 9:** Multivariate mixed effects generalized repeated measures analysis explaining the effect of the educational intervention program on the nurses perception toward parental participation. N = 320 records.

Parameter	Estimate	Std. Error	df	t	Sig.	95% Confidence Interval	
						Lower Bound	Upper Bound
Intercept	8.237	.1403	316	58.6	.000	7.9	8.513
[Group = intervention]	.675	.198	316.000	3.401	.001	.2844	1.065
[Group = control reference comparison]	0	0	.	.	.	.	.
[time1 = post-test	1.100	.198	316.000	5.5	.000	.709	1.490517
[time2 = pre- test]	0^b^	0	.	.	.	.	.
[Group = intervention] * [time1 = post-test]	-0.912	.280	316	-3.2	.001	-1.464	-.3602
[Group = intervention] * [time2 = pre-test]	0^b^	0	.	.	.	.	.
[Group = control] * [time1 = post-test]	0^b^	0	.	.	.	.	.
[Group = control] * [time2 = pre-test]	0^b^	0	.	.	.	.	.

## Discussion

The findings expressed that the educational group was statistically different in knowledge after applying to the KMC educational program. Moreover, the findings expressed that the educational group was statistically different in attitude after the educational program. Also, the results reflect a significant effect in the educational group of perceptions of parental participation. AlMutairi et al. [28], conducted a study in the UK involving 68 NICU nurses, and showed that knowledge levels significantly increased after ongoing education about KMC. A further two studies were conducted in the United Kingdom regarding the impact of educational programs on KMC among NICU nurses [29, 30].

Firstly, the study by Cooper et al. [29] showed that knowledge and attitudes about KMC improved significantly after intervention. Secondly, Kwah et al. [30] showed that knowledge about KMC significantly increased after the program. Similarly, Hendricks-Munoz and Mayers [31] conducted a study of ICU nurses working in Bellevue Hospital Center in New York. The findings showed that (16.7%) of nurses were unable to perform KMC in stable infants’ condition, (66%) of nurses were uncomfortable when performing KMC on neonates with NCPAP, and (90%) reported feelings of discomfort when the neonate was on a ventilator. However, post-training findings expressed an improvement in skill competency when the same nurses practiced KMC on neonates on CPAP (from 30% to 90%) and ventilator (from 10% to 48%). The feelings of discomfort when dealing with neonates on nasal cannula, CPAP, and ventilator decreased to 0% which clearly demonstrated that education improved the value of KMC.

Similarly, two studies were conducted in Egypt regarding the impact of educational programs on KMC among NICU nurses [32, 33]. Firstly, Lawend et al. [32] showed that education and training courses had a positive effect in promoting knowledge and attitudes toward KMC. Secondly, Mohamed et al. [33] showed highly significant differences between pre- and post- education for KMC knowledge. Similarly, Samsudin et al. [34] conducted a study in Malaysia and used a modified KMC questionnaire that showed that knowledge levels significantly increased post-intervention, and the post-test knowledge score (M = 46.81, SD = 3.21) taken after the intervention program was significantly higher than the pre-test score. Regarding perceptions of the importance of family participation, Toivonen et al. [35] conducted a study in Finland that showed the highest nursing responses were found for active listening, parents’ trust in nurses, and emotional support. The authors emphasize the importance of including active listening skills in education interventions for KMC practices.

The findings showed that the nurses in the educational group had a significantly higher mean perception towards knowledge of KMC in general (both pre- and post- attitude combined) compared to those nurses in the non-educational group. The change in knowledge was statistically significant in the nurses who had received the intervention, their pre-test (post, as well as overall perception) exceeded that of those nurses who did not receive the educational session, which suggests a significant positive effect of the educational session on the knowledge of nurses in general. In the Middle East, Al-Shehri and Binmanee [36] conducted a study from Riyadh, Saudi Arabia. They found that many factors predicted knowledge of KMC. Our search uncovered four studies conducted in Africa regarding knowledge of KMC among nurses [1, 4, 37, 38]. Firstly, Ayele et al. [1], working in Ethiopia, conducted a study which emphasized that family support, availability of another caregiver at home, and maternal knowledge of KMC, which was considered as a prognostic factor to practice home KMC, are desirable predictors of KMC after being discharged. Secondly, the results of a study by Getie [37] revealed that educational level, place of residence, place of delivery, maternal knowledge, and support from family members, are the major predictors of KMC implementation.

In our study, the findings showed that the nurses in the educational group had a significantly higher mean attitude in general (both pre- and post- attitude combined) compared to those nurses in the non-educational group. However, the change in nurses’ attitudes was statistically significant in the nurses who had received the intervention, their pre-test (post-, as well as overall attitude) exceeded that of those nurses who did not receive the educational session, which suggests a significant positive effect of the educational session on the attitudes of nurses in general.

Al-Shehri and Binmanee [36] in Saudi Arabia found that many factors predicted attitudes toward KMC. Similarly, we looked at four studies conducted in Africa regarding attitudes toward KMC among nurses [1, 4, 37, 38]. Firstly, Ayele et al. [1] in Ethiopia conducted a study that emphasized that family support, availability of another caregiver at home, and maternal knowledge of KMC were considered prognostic factors to attitudes toward KMC among nurses. Secondly, Getie [37] revealed that educational level, place of residence, place of delivery, maternal knowledge, and support from family members, are the major factors that determine knowledge and attitudes toward KMC. Kassaw et al. [4] found that the major factor impacting knowledge and attitude towards KMC practice is wealth. Fourthly, Lydon et al. [39] conducted a study in Malawi and found that social support, community support, and economic support were predictors of attitude toward KMC among nurses in NICU.

The findings showed that the nurses in the educational group had a significantly higher mean perception towards parental participation in general (both pre- and post- perception combined) compared to those nurses in the non-educational group. However, the change in nurses’ perceptions was statistically significant in the nurses who had received the intervention, since their pre-test (post-, as well as overall perception) exceeded that of those nurses who did not receive the educational session, which suggests a significant positive effect of the educational session on the nurses’ perceptions in general. Kassaw et al. [4] found that the factors that impact nurses’ perceptions of parental participation were social support, community support, and economic support. In Ethiopia, Yusuf et al. [38] revealed that women who had a normal vaginal delivery are 7 to 8 times more likely to utilize KMC than women who delivered by cesarean section. Women who were consistently exposed to KMC practices when their infant was in the NICU were more likely to continue KMC when they were discharged and returned home. This may be considered a highly determining factor for perceptions of parental participation in NICU nurses, alongside the time of KMC initiation, which was statistically estimated [18, 40].

### Limitations

This study encountered few limitations that can impact the study. The scheduling of the educational intervention was considered as another limitation, the authors tried to schedule the sessions based on the best time of the nurse’s working hour. Also, the study used quasi-experimental design which is not the best design. However, the time and schedule of the nurses duties did not give the opportunity to apply randomized controlled design.

### Implications

The results of our study offer evidence-based information to healthcare providers that reflect on their clinical practices and application of competencies (a healthcare provider is unable to practice what they don’t know). These interventions can translate to health care and lead to important changes in the parental support generally available in Jordan, which is to include education in the routine of care. Also, nurses can develop their problem-solving and critical thinking skills which will serve to improve their practice in different situations. Policy makers need to focus on developing family-centered care by involving parents in practice, and fostering collaboration and shared decision-making among all healthcare providers, and especially nurses.

### Recommendations for future studies

More studies are required involving additional hospitals in Jordan and other health care teams in the study. The authors also recommend conducting further studies using a more powerful research design such as a time series design, for assuring the outcome after the information is kept, or through using qualitative design that can direct us to other significant points and outcomes and providing a multi days educational intervention program that is not just a full-day intervention. The design of this study and educational intervention must be reconsidered in the research that can be conducted in future. Session, emulation, and case study are valuable in nurses parental support education.

## Conclusion

Our study has shown that providing education on KMC increased nursing knowledge and consequently improved attitudes. Enhanced parental support from nurses and other healthcare providers can raise a parent’s level of satisfaction and decrease their negative feelings to achieve a high quality of neonatal care. The design of this study and educational intervention must be reconsidered in the research that can be conducted in future. Session, emulation, and case study are valuable in nurses parental support education.

## Supporting information

S1 File DATA KANGRO SA (1). (XLSX)
